# Communication of suicide intent by schizophrenic subjects: data from the Queensland Suicide Register

**DOI:** 10.1186/1752-4458-1-6

**Published:** 2007-10-31

**Authors:** Diego De Leo, Helen Klieve

**Affiliations:** 1 Australian Institute for Suicide Research & Prevention, Griffith University, Mt. Gravatt Campus, Queensland, 4111, Australia

## Abstract

**Background:**

Suicide in mentally ill subjects, like schizophrenics, remains unbearably frequent in Australia and elsewhere. Since these patients are known to constitute a high-risk group, suicide in them should be amongst the most preventable ones. The objective of this study is to investigate the frequency of suicide communication in subjects with reported history of schizophrenia who completed suicide.

**Method:**

The Queensland Suicide Register (QSR) was utilised to identify suicide cases. Frequency of suicide communication was examined in subjects with schizophrenia, and compared with persons with other psychiatric conditions and with subjects with no reported diagnosis. Socio-demographic variables, history of suicidal behaviour, pharmacological treatment and mental health service utilisation were also compared among the three groups.

**Results and discussion:**

Subjects with a reported diagnosis of schizophrenia comprised 7.2% (n = 135) of the 1,863 suicides included in this study. Subjects with schizophrenia and those with other psychiatric disorders communicated their suicide intent more frequently than those with no psychiatric diagnosis, and persons with schizophrenia communicated their intent more than those with other psychiatric diagnoses. Seventy one per cent of schizophrenia subjects had contact with a mental health professional within the three months prior to suicide.

**Conclusion:**

The fact that subjects with schizophrenia had the highest prevalence of suicide intent communication could offer concrete opportunities for suicide prevention.

## Background

Among patients with psychotic disorders, suicide is the major cause of premature death [1]. Between 10 and 15% of patients with schizophrenia would eventually die by suicide [2], and rates of suicide among them have been reported as increasing [3]. Lifetime suicide attempts are common in 18–55% of individuals [4]. Approximately 2–12% of all suicides are attributable to schizophrenia [5], and in studies investigating samples of inpatient suicides, as many as 76% of cases have a diagnosis of schizophrenia [6].

Suicide and suicide attempts among individuals with schizophrenia often result in a significant psychological, social and financial burden upon individuals and families. For suicide attempts, there is a considerable cost to the community associated with hospital care, treatment and rehabilitation. In Australia, for example, the estimated cost associated with suicide and self-inflicted injury directly attributable to schizophrenia in 2001 was an estimated $6 million, including $4 million in hospital costs [7].

The elevated lifetime risk of suicide completions and attempts in subjects with schizophrenia has been associated with particular risk factors, including social and demographic factors, aspects of symptomatology, co-morbid psychopathology, and characteristics of the course of illness [8-10]. Interestingly, it has been suggested that individuals with schizophrenia are less prone to communicate suicidal intent to others than persons with other psychiatric disorders [11,12]. As a result, suicidal behaviours among patients with schizophrenia and schizoaffective disorder are often considered to be less predictable than those made by patients without these disorders and completed suicide is often unexpected [12]. So far, only one Finnish study has dealt with the issue of communication of suicide intent in schizophrenic subjects [13]. This fact is in itself surprising, given the opportunities for suicide prevention that early detection of suicidality would provide in such a high-risk group of subjects.

The purpose of this investigation is to provide an account of the frequency of suicide communication among completed suicide cases with a reported diagnosis of schizophrenia, and to make a comparison with groups of people without history of such a condition.

## Method

A state-wide suicide register was utilised to conduct a retrospective study of all deaths in Queensland, Australia, which occurred from 1994 to 1998. Completed suicides obtained from the Queensland Suicide Register (QSR) were investigated.

Data contained in the QSR were derived from sources including a post-mortem examination report, toxicological results, *Report Concerning Death by Member of the Police Service (Form 4)*, and *Report to the Coroner by Police Officer in the Event of a Possible Suicide (Psychological Autopsy Report)*. The latter involves a structured interview developed by the Australian Institute for Suicide Research and Prevention in consultation with the Queensland Police Service and Coroners and revised periodically. These questionnaires are completed by specifically trained Police Officers, and usually involve close proxies. The interview on average lasts 90 minutes, and in most cases is performed within 2–10 days from the discovery of the body.

Specific inclusion criteria were devised for the study; subjects were considered to have been suffering from schizophrenia if: 1) the deceased had a diagnosis of schizophrenia or other psychotic disorder (according to ICD 9 and 10 criteria) prior to death; or 2) in cases in which the available documents referred diagnoses such as "chronic psychosis", "paranoia", "acute paranoia", "dissociative psychosis", "delusional state", "chronic delusions", there was evidence of a long-term (>6 months) treatment with a neuroleptic drug prior to death; 3) the death was accompanied by a completely filled-in psychological autopsy report; and 4) it was 'beyond reasonable doubt' that the death was attributable to suicide. Due to the law protecting privacy, it was not possible to check for any supplementary material (including clinical cards) in addition to the one provided by Coroner offices.

Comparison groups were selected on the basis that they met criteria 3 and 4 (above). The first comparison group (Group1) included suicides where the deceased had evidence of a psychiatric diagnosis distinct from schizophrenia [e.g. bipolar disorders, depressive disorders (inclusive of psychotic features), anxiety disorders, substance-related disorders etc)]. The second comparison group (Group2) comprised suicides where the deceased did not have any identified psychiatric diagnoses/psychiatric contact prior to death.

Previous suicide communications and threats were the main object of this investigation. For the purposes of the study, a suicide threat was defined as 'any communication that indicates a desire to end one's life prematurely', whilst a suicide attempt was defined as 'any non-habitual act of self-harm with or without ascertainable intent to die' *(from the Psychological Autopsy Report)*.

Socio-demographic variables investigated included age, gender, marital status, employment status, and living arrangement. Other variables included suicide method, co-morbid psychopathology, inpatient/outpatient treatment, prescription of psychiatric medications, and use of mental health services. Ethical clearance was obtained through Griffith University Ethics Committee.

Chi-square tests were used to profile the sample, compare the three diagnostic groups and also to assess factors that may differentiate individuals with schizophrenia from other suicide cases in the two comparison groups (Group1 and Group2). Statistically significant differences were reported using p < 0.05. Odds ratios were calculated to provide a measure of the impact in appropriate situations. The statistical software utilised for the data analysis was SPSS version 14 for Windows.

## Results

A total of 1,863 suicides met the defined inclusion criteria. Of these, subjects with reference to a diagnosis of schizophrenia comprised 7.2% (n = 135) of all cases (inclusive of 19 cases meeting selection criterion #2), whilst other diagnosed psychiatric disorders comprised 28.9% (n = 538), and cases with no reported presence of any psychiatric condition constituted 63.9% (n = 1,190). Thus, in total, for only 36.1% of all suicides there was evidence in coroners' documents of a psychiatric disorder, a percentage remarkably lower than that reported in most studies [14]. Socio-demographic variables are reported in Table 1.

**Table 1:** Socio-demographic characteristics of subjects with a diagnosis of schizophrenia, with a diagnosis of other psychiatric conditions, and without any psychiatric diagnosis (Queensland Suicide Register)^#^

	Subjects with Schizophrenia	Subjects with Other Psychiatric Conditions (Group1)	Subjects with No Psychiatric Diagnosis (Group2)
	n (%)	n (%)	n (%)

**Gender**	(n = 135)	(n = 538)	(n = 1190)
Male	106 (78.5)	372 (69.1)	1030 (86.6)
Female	29 (21.5)	166 (30.9)	160 (13.4)
Group Comparisons ^##^	*χ*^2 ^(2) = 73.36 ***	*χ*^2 ^(1) = 4.61 * OR = 1.63 (1.04, 2.56)	*χ*^2 ^(1) = 6.40 * OR = .57 (0.37, 0.88)
**Age (years)**	(n = 135)	(n = 538)	(n = 1190)
0–24	35 (25.9)	66 (12.3)	283 (23.8)
25–34	43 (31.9)	101 (18.8)	294 (24.7)
35–44	29 (21.5)	147 (27.3)	235 (19.7)
45–54	19 (14.1)	111 (20.6)	134 (11.3)
55+	9 (6.7)	113 (21.0)	244 (20.5)
Average	34.20 (100)	43.26 (100)	39.34 (100)
Median (Inter-Quartile Range)	32 (18)	42.00 (20)	35 (24)
Group Comparisons ^##^	*χ*^2 ^(8) = 80.83 ***	*χ*^2 ^(4) = 38.09 ***	*χ*^2 ^(4) = 15.84 **
**Marital Status**	(n = 131)	(n = 510)	(n = 1114)
Never married	77 (58.8)	125 (24.5)	428 (38.4)
Married/de facto	23 (17.6)	204 (40.0)	360 (32.3)
Separated	13 (9.9)	94 (18.4)	171 (15.4)
Divorced	16 (12.2)	64 (12.5)	93 (8.3)
Widowed	2 (1.5)	23 (4.5)	62 (5.6)
Group Comparisons ^##^	*χ*^2 ^(8) = 72.80 ***	*χ*^2 ^(4) = 60.57 ***	*χ*^2 ^(4) = 28.33 ***
Never Married/Other (unknown excluded)		*χ*^2 ^(1) = 56.71 *** OR = 4.39 (2.94, 6.57)	*χ*^2 ^(1) = 20.15 *** OR = 2.29 (1.58, 3.30)
**Living Arrangements**	(n = 131)	(n = 516)	(n = 1105)
Spouse/friend/Relative	41 (31.3)	259 (50.2)	554 (50.1)
Parents	42 (32.1)	67 (13.0)	185 (16.7)
Other shared/institutional	11 (8.4)	31 (6.0)	26 (2.4)
Alone/homeless	37 (28.2)	159 (30.8)	340 (30.8)
Group Comparisons ^##^	*χ*^2 ^(6) = 51.30 ***	*χ*^2 ^(3) = 31.76 ***	*χ*^2 ^(3) = 38.14 ***
Living with Parents/Other living arrangements (unknown excluded)		*χ*^2 ^(1) = 27.14 *** OR = 3.16 CI: (2.02, 4.95)	*χ*^2 ^(1) = 18.33 *** OR = 2.35 CI: (1.57, 3.50)
**Employment Status**	(n = 131)	(n = 526)	(n = 1120)
Employed	29 (22.1)	205 (39.0)	536 (47.9)
Unemployed	46 (35.1)	127 (24.1)	287 (25.6)
Disabled/retired	22 (16.8)	108 (20.5)	192 (17.1)
Not in labour force	34 (26.0)	86 (16.3)	105 (9.4)
Group Comparisons ^##^	*χ*^2 ^(6) = 62.03 ***	*χ*^2 ^(3) = 19.18 ***	*χ*^2 ^(3) = 50.17 ***
Not in Employment/Employed (unknown excluded)		*χ*^2 ^(1) = 12.96 *** OR = 2.25 (1.44, 3.52)	*χ*^2 ^(1) = 31.33 *** OR = 3.23 (2.10, 4.95)

^# ^Sample sizes for each variable indicated, with unknown cases not included in individual analyses

^##^*Comparisons provided for differences between the 3 diagnostic groups (column 2) and for comparison Group1 and Group2(columns 3 and 4)*. Significance: * p < .05, ** p < .01, *** p < .001. Odds Ratios (confidence interval) provide the strength and direction of the relationship, indicating the liklihood of subjects with schizophrenia being more (>1) or less likely (<1) than those with other psychiatric disorders (Column3) or those with no identified disorder (Column 4) to exhibit the charactristic under consideration (ie having never married, living with parents, or not being in employment).

In firstly comparing the three diagnostic groups for socio-demographic variables, there were highly significant differences observed. Age at the time of death differed across diagnostic groups [χ^2^(8) = 80.83, p < .001], with subjects with schizophrenia being significantly younger than either comparison group. There were significant differences in gender [χ^2^(2) = 73.36, p < .001], marital status [χ^2^(8) = 72.80, p < .001], living arrangements [χ^2^(6) = 51.30, p < .001] and in employment status [χ^2^(6) = 62.03, p < .001]. Overall, compared to groups 1 and 2, subjects with schizophrenia were more likely to be younger, to have never been married, to have been living with parents, and to have been unemployed at the time of death. With regard to gender, while there were significant differences between those with schizophrenia and individuals in Group1 and Group2 [χ^2^(1) = 4.61, p < .05 and χ^2^(1) = 6.40, p < .05], the highest proportion of males was seen in Group2 (86.6%) (Tab. 1).

Across all diagnostic groups, the majority of individuals had threatened to commit suicide at some point in time throughout their life. However, there was a significant difference between the three groups on frequency of communication of suicide intent and previous suicide attempts. In these two aspects, the groups significantly differed both for the past year and lifetime (Table 2).

**Table 2:** Suicide communication/threats, attempts and methods in each diagnostic group^#^

	Subjects with Schizophrenia	Subjects with Other Psychiatric Conditions (Group1)	Subjects with No Psychiatric Diagnosis (Group2)
	n (%)	n (%)	n (%)

**Lifetime communication of suicidal intent**			

	(n = 108)	(n = 419)	(n = 808)

Yes	90 (83.3)	322 (76.8)	433 (53.6)
No	18 (16.7)	97 (23.2)	375 (46.4)
*Group Comparisons*^##^	*χ*^2 ^(2) = 84.57 ***	*χ*^2 ^(1) = 2.12 ns OR = 1.51(0.87,2.62)	*χ*^2 ^(1) = 34.41 *** OR = 4.33 (2.56,7.32)

**Communication of suicidal intent 12-months prior**			

	(n = 111)	(n = 429)	(n = 847)

Yes	85 (76.6)	278 (64.8)	391 (46.2)
No	26 (23.4)	151 (35.2)	456 (53.8)
*Group Comparisons*^##^	*χ*^2 ^(2) = 63.88 ***	*χ*^2 ^(1) = 5.55 * OR = 1.78 (1.10, 2.88)	*χ*^2 ^(1) = 36.31 *** OR = 3.81 (2.41,6.04)

**Lifetime Suicide Attempt/s**			

	(n = 119)	(n = 487)	(n = 969)

Yes	65 (54.6)	254 (52.2)	218 (22.5)
No	54 (45.4)	233 (47.8)	751 (77.5)
*Group Comparisons*^##^	*χ*^2 ^(2) = 151.01 ***	*χ*^2 ^(1) = 0.23 ns OR = 1.10 (0.74,1.65)	*χ*^2 ^(1) = 56.83 *** OR = 4.15 (2.80,6.13)

**Suicide Attempt/s 12-months prior**			

	(n = 118)	(n = 482)	(n = 972)

Yes	52 (44.1)	178 (36.9)	158 (16.3)
No	66 (55.9)	304 (63.1)	814 (83.7)
*Group Comparisons*^##^	*χ*^2 ^(2) = 99.87 ***	*χ*^2 ^(1) = 2.04 ns OR = 1.35 (0.90,2.02)	*χ*^2 ^(1) = 52.33 *** OR = 4.06 (2.72,6.06)

**No of Co-morbid Conditions**			

	(n = 135)	(n = 538)	(n = 0)

One or more	39 (28.9)	62 (11.5)	-
None	96 (71.1)	476(88.5)	-
*Group Comparisons*^##^		*χ*^2 ^(1) = 25.51 *** OR = 3.12 (1.98, 4.92)	

**Suicide Method**			

	(n = 131)	(n = 526)	(n = 1120)

Drugs/poison	26 (19.3)	127 (23.8)	120 (10.2)
MV carbon monoxide	14 (10.4)	103 (19.3)	248 (21.0)
Hanging	45 (33.3)	154 (28.8)	437 (37.1)
Firearms	15 (11.1)	66 (12.4)	275 (23.3)
Jumping/lying before moving object	8 (5.9)	8 (1.5)	13 (1.1)
Other	27 (20.0)	76 (14.2)	86 (7.3)
*Group Comparisons*^##^	*χ*^2 ^(10) = 139.21 ***	*χ*^2 ^(5) = 17.90 **	*χ*^2 ^(5) = 64.92 ***

^#^Sample sizes for each variable indicated, with unknown cases not included in individual analyses.

^## ^Comparisons provided for differences between the 3 diagnostic groups (column 2), and for comparison Group1 and Group2 (columns 3 and 4). Significance: * p < .05, ** p < .01, *** p < .001. Odds Ratios (confidence interval) provide the strength and direction of the relationship, indicating the liklihood of subjects with schizophrenia being more (>1) or less likely (<1) than those with other psychiatric disorders (Column3) or those with no identified disorder (Column 4) to exhibit the charactristic (ie suicidal ideation or attempt or the presence of co-morbid conditions).

In the year before death, schizophrenic subjects communicated their intent to die 1.78 times more frequently than individuals who suffered from other psychiatric conditions [n = 540, χ^2^(1) = 5.55, p < .05, OR = 1.78, CI: 1.10, 2.88]. However, a significant difference was observed between individuals with schizophrenia and Group1 with regard to living arrangements [n = 647, χ^2^(3) = 31.76, p < .001] and employment status [n = 657, χ^2^(3) = 19.18, p < .001], with more schizophrenics than subjects with other diagnoses living with parents and being unemployed. It might be hypothesised that these two conditions could have favoured a more frequent reception of suicidal messages by people cohabiting with the schizophrenic subjects.

There was also a significant difference in the number of persons who suffered one or more co-morbid psychiatric conditions [n = 673, χ^2^(1) = 25.51, p < 0.001, OR = 3.12, CI: 1.98, 4.92], with individuals with schizophrenia 3.12 times more likely to have suffered from one or more co-morbid psychiatric conditions (mainly depression and drugs/alcohol abuse) than those with other psychopathologies. In assessing patterns of communication of suicide against the presence of co-morbid diagnoses it was found that, in schizophrenic cases, there was a significant difference in suicide attempts both over a lifetime [n = 119, χ^2^(1) = 10.15, p < .01 OR = 4.08 CI: 1.66, 10.00] and in the last twelve months [n = 118, χ^2^(1) = 8.25, p < .01 OR = 3.30 CI: 1.44, 7.58] with a higher level of attempts in those with co-morbid conditions. This pattern was not significant in those with other psychopathologies.

The majority of persons with a diagnosis of schizophrenia (71.1%, n = 96) were in contact with a mental health professional within the three months leading up to suicide. In 20% (n = 28) of cases it was not known or unclear whether a mental health professional was consulted during this period. Five cases (3.7%) were inpatients at the time in which the suicide occurred.

## Discussion

Despite much advancement in psychiatry and related sciences, schizophrenia continues to be associated with an unbearably high risk of suicide [3]. In the current study subjects with evidence of such a diagnosis accounted for 7.2% of cases, a proportion that is comparable to previous psychological autopsy studies [5,15]. While confirming a number of characteristics related to socio-demographics, clinical features and suicide methods (Tab. 2) already known in the international literature, this study based on an Australian sample has reinforced a similar, initial observation from the National Suicide Prevention Project in Finland which reported on the three-month period preceding death [13]. However, in this experience, not only did subjects with schizophrenia frequently communicate their intention to die, but they did so more often than those with other psychiatric conditions. It is possible that the communication of suicide intent in the group with schizophrenia may be more predictive of completed suicide than in other psychiatric conditions, and much more so than in cases without a diagnosis. Surely the socio-demographic characteristics of the group of schizophrenics are relevant to the frequency of communication observed. In fact, this group represent a younger population with a presumably greater prevalence of interactions with "significant others", particularly parents, through living arrangements (over 32% living with parents and over 58% never having married) and, due to lesser levels of employment (over 77% currently not in employment), more time in the home environment.

Evidence of suicidality appeared most marked during the 12 months preceding death, both in terms of communication of intent and suicide attempt(s). At theoretical level, this should greatly increase the opportunities for intercepting suicidal trajectories and eventually preventing suicide in subjects affected by schizophrenia. However, despite the frequency in those subjects of suicide communication and attempts, both proxies and health care providers might find it difficult to perceive the level of suicidality that a person with such a diagnosis expresses. Maybe in these patients suicide communications and behaviours are too readily accepted as manifestations of a psychotic condition, and considered in the same way as hallucinations, delusions, and bizarre behaviours are. This could have a significant bearing upon the way in which suicide risk is approached and managed within treatment settings.

Attention to the detection and treatment of concomitant psychiatric conditions, particularly mood disorders [9] and drugs and alcohol abuse [2,16], may further provide indication of suicide risk and give additional opportunities for suicide prevention. This appears to be particularly important from the present study, since people with a diagnosis of schizophrenia were 3.12 times more likely to have one or more co-morbid disorders than persons with other psychiatric diagnoses. However, it has been reported that co-morbid substance abuse might decrease the likelihood of active aftercare [16]. Nevertheless, in the present experience, almost three quarters of schizophrenic subjects were in contact with a mental health professional in the last three months of their lives, a fact that should have facilitated appropriate intervention and consequently suicide prevention.

A final observation concerns the gender distribution of suicide, which in this experience is much more illustrative of the general population than that reported in the literature for schizophrenia (for example[17]). In fact, the male to female ratio was greater than 3 to 1, and thus, very similar to that of the general population of suicides in Queensland [18].

The present study has several limitations. First, information concerning suicide victims is based on retrospective, subjective information provided by family members, relatives or friends, as well as attending health care personnel. As a result, the validity and reliability of the data may be questionable. Due to both the complex nature of bereavement following suicide and the recollection of emotional events and relationships, information obtained about the close relative or friend may be subject to recall bias [19,20].

In cases where the diagnosis of schizophrenia was not clearly reported but a diagnosis suggestive of it was present (eg. "chronic psychosis" etc), evidence of a pharmacotherapy with a neuroleptic for at least 6 months was used as inclusion criterion. Following this opportunistic decision, 19 individuals were included in the study and 7 were excluded. However, it is important to notice that only prevalence data would be affected if these subjects were to be removed from data analysis. All other results would maintain their significance.

A further limitation of the study relates to the fact that some individuals may have completed suicide amidst a first psychotic episode. In this case, family or friends may not have recognised or been informed by the individual of the onset of symptoms. The potential for a diagnosis to be made would have also depended upon whether or not the individual presented him/herself to a mental health or general practitioner at the time of illness onset [21]. As a result, a number of suicide victims registered in the QSR might not have received appropriate diagnosis. In addition, the structure of the psychological autopsy adopted in the QSR permits only a certain level of identification of psychiatric disorders. This may affect the prevalence of psychiatric disorders within the QSR and eventually explain the low prevalence reported in this study (36.1%). However, from further work being undertaken, this factor appears to have less impact on the diagnosis of schizophrenia than other psychiatric diagnoses such as unipolar depression or anxiety disorders. A quality control evaluation of over 100 QSR cases with the use of SCID [22] by trained psychologists has ascertained the same proportion of schizophrenic subjects described in this paper (7%) (De Leo et al, in preparation). However, the effect of this limitation will be a slight undercounting of identified schizophrenics, and also, potentially, the inclusion of a small number in Group1 and Group2 – thus the final assessments reported will in fact understate the true difference between these groups.

Finally, this study focuses on data of completed suicides without comparing patterns of communication in a control group including both other psychiatric conditions and individuals with no diagnosis where suicide did not occur. The absence of such data imposes a particular limitation of not being able to assess the link between such communications and subsequent suicide, and in particular not being able to assess the likelihood of false positives. Nevertheless, the data used in this study suggest that, contrary to the general view that there is limited prior communication of suicidal intent by schizophrenic patients, such communication is frequent and could provide a greater capacity for more targeted intervention for suicide prevention with the necessary recognition that there will be instances where "false" expressions of intent will be made.

## Conclusion

The preventability of suicide among individuals with schizophrenia could be facilitated by a better detection of the explicit suicidal indications that these subjects may provide. These include a frequent history of suicide attempts and a common tendency for such persons to communicate their suicidal intent to others, particularly during the year preceding death. At present, it is possible that these important signals are not appropriately interpreted, or that a sort of clinical "blind eye" attitude tends to overlook such overt communications.

## Competing interests

The author(s) declare that they have no competing interests.

## Authors' contributions

Diego De Leo has conceived and designed the study. Helen Klieve has contributed with data analysis. Both have participated in the writing of the article and have read and approved the final manuscript.
